# Coronary calcium quantification using contrast‐enhanced dual‐energy computed tomography scans

**DOI:** 10.1120/jacmp.v14i3.4014

**Published:** 2013-05-06

**Authors:** Didem Yamak, William Pavlicek, Thomas Boltz, Prasad M. Panse, Metin Akay

**Affiliations:** ^1^ Systems of Biological and Health Systems Engineering Ira A. Fulton Schools of Engineering, Arizona State University Tempe AZ USA; ^2^ Department of Radiology Mayo Clinic Arizona Scottsdale AZ USA; ^3^ Department of Radiology Mayo Clinic Arizona Phoenix AZ USA; ^4^ Department of Biomedical Engineering Cullen College of Engineering, University of Houston Houston TX USA

**Keywords:** calcium, computed tomography, Agatston, volume score, dual energy

## Abstract

The purpose of this study is to evaluate a direct measure of calcium burden by using dual‐energy computed tomography (DECT) during contrast‐enhanced coronary imaging, potentially eliminating the need for an extra noncontrast X‐ray acquisition. The ambiguity of separation of calcium from contrast material on contrast‐enhanced images was solved by using virtual noncontrast images obtained by DECT. A new threshold CT number was required to detect the calcium carrying potential risk for adverse coronary events on virtual noncontrast images. Two methods were investigated to determine the 130 HU threshold for DECT scoring. An *in vitro* anthropomorphic phantom with 29 excised patient calcium plaques inserted was used for both a linear and a logistic regression analysis. An IRB approved *in vivo* prospective study of six patients was also performed to be used for logistic regression analysis. The threshold found by logistic regression model to define the calcium burden on virtual noncontrast images detects the calcium carrying potential risk for adverse coronary events correctly (2.45% error rate). DECT calcium mass and volume scores obtained by using the threshold correlates with both conventional Agatston and volume scores (r=0.98,p<0.001). A conventional CT cardiac exam requires two scans, including a noncontrast scan for calcium quantification and a contrast‐enhanced scan for coronary angiography. With the ability to quantify calcium on DECT contrast‐enhanced images, a DECT cardiac exam could be accomplished with one contrast‐enhanced scan for both calcium quantification and coronary angiography.

PACS numbers: 87.57.Q, 87.57.N

## I. INTRODUCTION

Large amounts of calcium plaque in the coronary arteries is a known independent risk factor for adverse coronary events.^1^, ^2^, ^3^ Conversely, a negative coronary calcium scan has a high predictive value for reduced risk of death from coronary artery disease (CAD). Noncontrast CT is the established and routine test for noninvasive detection of coronary artery calcium (CAC). Conventional coronary CT angiography (CCTA) using iodine contrast for vascular evaluation is frequently acquired in addition to a noncontrast scan. Recently, DECT coronary angiography has been performed clinically.^(4)^ DECT has the ability to generate virtual monochromatic energy images, as well as material basis pair images. DECT‐derived monochromatic X‐ray images suffer far less from the blooming artifact, so that large calcium plaques may not interfere with assessment of residual lumen. Patients needing evaluation for in‐stent restenosis also are difficult to visualize with conventional CCTA. The blooming artifact from stents can be removed with DECT (virtual nonstent images). The patient population for which CCTA is appropriate could potentially expand if the presence of stents or large amounts of calcium is no longer an impediment for DECT. However, concern may exist for added radiation dose with DECT. A conventional CCTA coupled with noncontrast CACs is about 2.8mSv. A single fast‐switched DECT is approximately the same at 2.7mSv without the extra noncontrast acquisition.^(4)^ Therefore, dose‐neutral DECT total exam can possibly be achieved if the noncontrast exam can be eliminated.

Agatston score and volume score are both used clinically in the assessment of risk in terms of calcification. Agatston scores are currently obtained using 120 kVp CT acquisitions without iodine contrast. Computation of the score uses a threshold whereby pixels greater than 130 Hounsfield units (HU) are classified as calcified tissue. For a given ‘plaque’ (composed of pixels more than 130 HU), the total area (mm^2^) is multiplied by a weighting factor that depends upon the ‘peak’ CT HU contained in the plaque region.^(5)^

To compute a conventional Agatston score S:

(1) S=(w)(Area of plaque)

with weighting factor (w) given by: 0 if ${CT}^{\max}<130\;HU$; 1 if $130\;HU\leq {CT}^{\max}<200\;HU$; 2 if $200\;HU\leq {CT}^{\max}<300\;HU$; 3 if $300\;HU\leq {CT}^{\max}<400\;HU$; 4 if $400\;HU\leq {CT}^{\max}$.

Plaque scores from all vessels are summed to calculate at a composite score. A total CAC score from all major arteries in the heart describes the extent of coronary artery disease. If the total CAC score is 0, there is no identifiable disease. If total CAC score is 1 to 99, mild disease is present. If the total CAC score is 100 to 399, moderate disease is present. If the total CAC score is greater than 400, severe disease is present.

Alternatively, a volume score is computed as:

(2) $$V=({\text{Volume}}_{\text{Voxel}})({\text{Number}}_{\text{Voxel}})$$

where ${\text{Number}}_{\text{Voxel}}$ is the number of calcified voxels. Volume scores use the 130 HU threshold for calcium identification, and attempt to quantify the total volume of calcification in the coronary vasculature.^6^, ^7^, ^8^

Multidetector computed tomography (MDCT) at 120 kVp, axial mode, and FOV of 25 cm is widely used for coronary calcium scoring due to the high correlation between MDCT and the original method of electron beam CT (EBCT).^9^, ^10^, ^11^, ^12^ MDCT with thin (2.5 to 3.0 mm) slices is commonly obtained using prospective gating, a technique that briefly irradiates (∼175 msec) during ventricular diastole of the R‐to‐R interval to decrease patient dose. In order to assess vessel patency, a second scan with iodinated contrast material is performed to delineate vessels and separate blood from myocardial tissue. Both acquisitions are obtained axially with thin slices and prospective gating. Image data are postprocessed to create curvilinear plane images that follow coronary vessel curvature and aid visualization of the entire vessel.

Commercially available DECT scanners achieve dual energy through two X‐ray sources, or two detector arrays, or a single tube equipped with fast voltage switching. Fast‐kVp‐switching DECT rapidly toggles (as short as 250 μsec) between 80 and 140 kVp, resulting in two datasets from the high‐ and low‐voltage energies.^(13)^ The acquisitions collect projection data that are nearly perfectly registered in both time and projection space.

DECT exploits the fact that different tissues have different mass attenuation coefficients when interacting with X‐rays of different energies. Specifically, a DECT scanner examines the patient using two different X‐ray energies in order to determine the energy dependency of tissue attenuation values for each voxel. Each voxel can be mathematically decomposed as a linear combination of the mass attenuation coefficients corresponding to any pair of materials. A constraint is applied to each individual voxel such that the combined material basis combination achieves the same X‐ray behavior as the tissues actually present in the voxel. These ‘basis materials’ are then individually depicted in the image once their mass fractions have been determined according to the published values of monochromatic X‐ray mass attenuation from the National Institute of Standards and Technology.^(14)^

Additionally, energy specific (keV) HU images (virtual monochromatic X‐ray images) can be calculated from fast‐kVp‐switched DECT as:

(3) $$\mu_{x}(E)=(\rho_{1})(f_{1})(\mu_{m1}(E))+(\rho_{2})(f_{2})(\mu_{m2}(E))$$

where $\mu_{x}(E)$ is the linear attenuation coefficient of the target material at specified keV, $\rho_{1}$ and $\rho_{2}$ are the densities of the basis materials 1 and 2, $f_{1}$ and $f_{2}$ are the mass fractions of basis materials 1 and 2, and $\mu_{m1}(E)$ and $\mu_{m2}(E)$ are the mass attenuation coefficients of basis materials 1 and 2. Virtual monochromatic images reduce beam hardening artifacts that would be present in conventional polychromatic images, but may not sufficiently separate calcium from iodine.^(15)^

Dual‐energy CT basis material images display mass density $\left(mg/{cm}^{3}\right)$ as the CT number.^(16)^ DECT acquisitions enable the reconstruction of material basis pair images chosen by the operator.^(17)^ Reconstructing the material basis pair images Calcium(Iodine) or Water(Iodine) produces virtual noncontrast images from the iodine contrast‐enhanced portion of a fast‐switched DECT. Calcium and iodine were selected as basis materials to allow for the creation of ‘virtual’ noncontrast calcium images in which calcium content is preserved in the image. This approach may provide a measure of calcium plaque burden that is comparable to true noncontrast CAC scoring.^(11)^ The purpose of this study is to evaluate a direct measure of calcium burden by using DECT during contrast‐enhanced coronary imaging, potentially eliminating the need for an extra noncontrast X‐ray acquisition. Measured calcium amounts by contrast‐enhanced DECT scans were also compared to clinically used Agatston score and volume scores obtained by noncontrast single‐energy CT scans.

## II. MATERIALS AND METHODS

Both dual‐energy and conventional single‐energy coronary imaging were performed using a General Electric (GE) Discovery HDCT750 (GE, Waukasha, WI). This unit was equipped with fast voltage switching and Gemstone Spectral Imaging (GSI) cardiac protocols. All conventional CT coronary acquisitions were 120 kVp, while DECT switched between nominal 80 kVp and 140 kVp during each projection. Postprocessing of conventional and GSI data was performed using a GE Advantage Windows (AW) (GE) workstation equipped with standard Agatston and volume scoring tools. No software tools are commercially available that support Agatston and volume scoring using source data from dual energy acquisition. An in‐house MATLAB (The MathWorks, Natick, MA) program was utilized for volume and calcium mass scoring with DECT datasets. For consistency, conventional CT calcium scores were also generated using a MATLAB program. The AW provides a suite of GSI processing tools for generation of monochromatic energy image data, and also provides operator selectable material basis pairs for material basis image creation and clinical viewing (DICOM compatible). Material basis pair images were created for Water(Iodine), Iodine(Water), Calcium(Water), Water(Calcium), Calcium(Iodine), and Iodine(Calcium). By convention, an image identified as Calcium(Iodine) displays the calcium content from a calcium and iodine basis pair. Calcium and iodine were compelling choices as basis materials since those materials provide a meaningful description of the materials present when attempting to evaluate calcium during the peak enhancement phase of coronary artery imaging.

In order to calibrate DECT iodine‐enhanced data, several determinations were necessary. Firstly, the equivalent 130 HU threshold must be specified in equivalent material density units for Calcium(Iodine) image. Secondly, using the specified threshold, the calcium mass and volume scores were calculated on virtual noncontrast images and correlated to the Agatston and volume scores obtained by true noncontrast scans.

### A. Equivalent 130 HU threshold determination for Calcium(Iodine) image

Two different methods were performed to find the equivalent 130 HU threshold for DECT scoring.

**Figure 1:**
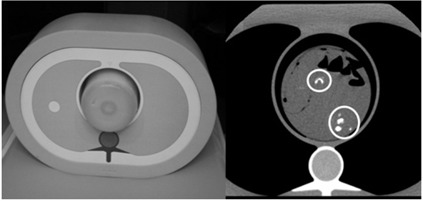
Phantom built with endarterectomy tissue samples: phantom containing endarterectomy tissue samples in 4% iodine:saline (left); axial slice at 120 kVp with circles around calcium plaque samples (right). All 29 calcium plaques from six patients were imaged.

The first method aimed to correlate DECT calcium density values with 120 kVp HU values using the pixel values recorded from Calcium(Iodine) and 120 kVp images of six anonymous *ex vivo* patient tissue samples (endarterectomy surgery specimens) which provided 29 calcified plaques. Six bags filled with patient plaques were surrounded by saline solution and inserted into a cardiac CT calibration phantom.^(6)^ The phantom, shown in Fig. 1, consists of an anthropomorphic chest section with artificial lung that mimics human tissue in density and attenuation characteristics, but the phantom was not capable of creating cardiac motion. All 29 patient calcium plaques were scanned with the prospective ECG triggering protocol used on patients. A cyclical ECG signal was simulated from scanner's ECG monitor in order to perform the triggering. The prospective ECG‐triggering protocol also used 16×2.5 mm collimation, 0.35 sec rotation, and pitch of 1. The plaques were scanned at the conventional 120 kVp and with fast‐switched DECT 80 kVp and 140 kVp. Images were reconstructed with a field of view (FOV) of 25 cm using cardiac standard kernel and image thickness and increment of 2.5 mm. Region of interest (ROI) was determined according to the edges of the calcium plaques in 120 kVp images and ROI locations were coregistered between conventional images and DECT images. Each pixel value ($mg/{cm}^{3}$ or HU value) within each plaque was recorded for both image types. Linear regression analysis was performed between conventional CT HU and Calcium(Iodine) $mg/{cm}^{3}$ data.

The second method applied was logistic regression, which is generally used to predict the occurrence probability of an event. Defining calcium burden within a contrast material containing coronary artery can be considered as a two‐class classification problem whereby one class is calcium and the other class is iodine‐enhanced blood. Predictor variables were chosen as the pixel values collected from Calcium(Iodine) images of calcified plaques and iodine enhanced blood. Previously mentioned 29 calcified plaques obtained from six anonymous *ex vivo* patients were used to define the predictor variables of calcium class. Note that the pixels corresponding to the pixels equal or higher than 130 HU on 120 kVp images were collected since 130 HU threshold specifying in equivalent material density units for Calcium(Iodine) image needed to be defined. The pixels to define the predictor variables of iodine‐enhanced blood class were collected from six patients whose IRB and HIPPAA approvals and patient written informed consents were obtained. All six patients received both conventional and noncontrast single‐energy CT scan coupled with contrast‐enhanced fast‐switched DECT scan of the coronary arteries. Noncontrast scans were prospectively triggered using 120 kVp, 750 mA, 0.35 sec rotation, 1.0 pitch, and 16×2.5 mm detector configuration. DECT scans were prospectively triggered using 80 kVp/140 kVp, 715 mA, 0.35 sec rotation, 1.0 pitch, and 64×0.625 mm detector configuration. DECT protocols were adjusted (increased mAs) for two larger patients and acquired with 765 mA and 0.6 sec rotation. Three patients received contrast enhancement with Omnipaque 350 (GE, Princeton, NJ) at $5\;{cm}^{3}/sec$, and the other patients received Visipaque 320 (GE, Princeton, NJ) at $4\;{cm}^{3}/sec$. Axial images were reconstructed at 75% of the R‐to‐R interval using a FOV of 25 cm and ‘standard’ kernel with image thickness and increment of 2.5 mm.

### B. Preliminary patient examinations: calcium scoring

**Figure 2:**
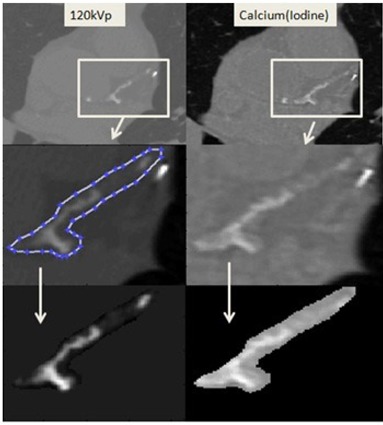
Vessel extraction on patient images. First Row: conventional 120 kVp noncontrast protocol (left); Calcium(Iodine) DECT (right) with iodine contrast administered (virtual noncontrast). Second Row: demonstration of vessel extraction (same boundaries are used while extraction is being done on Calcium(Iodine) images). Third Row: extracted vessels.

A total of 92 images obtained from six patients mentioned previously were used to extract the coronary arteries and the aorta. Both noncontrast single‐energy and contrast‐enhanced DECT acquisitions covered all calcified areas in the vessels, as given in Fig. 2. Agatston and volume scores were calculated for conventional CT data, volume and calcium mass scores were calculated for Calcium(Iodine) DECT image data. Calcium mass scores, $S_{\text{Mass}}$, were computed as:

(4) $$S_{\text{Mass}}=(\text{CT value})({\text{Volume}}_{\text{Voxel}})({\text{Number}}_{\text{Voxel}})$$

where ${Volume}_{Voxel}$ is the volume of one pixel, ${Number}_{Voxel}$ is the number of calcified pixels, and CT value is the recorded mean CT value of calcified area. Pixels having a CT value higher than the specified equivalent 130 HU threshold in material density units for Calcium(Iodine) image for DECT image data were averaged to find the mean CT value of the calcified area. Correlation was checked between volume scores obtained by 120 kVp and DECT Calcium(Iodine) image data, also between calcium mass scores obtained by DECT Calcium(Iodine) image data and Agatston scores obtained by 120 kVp image data.

## III. RESULTS & DISCUSSION

### A. Equivalent 130 HU threshold determination for Calcium(Iodine) image

**Figure 3:**
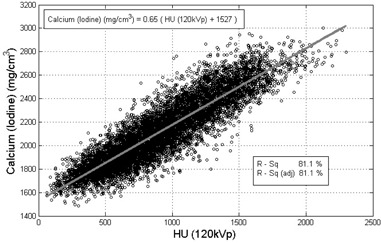
Recorded pixel values from 120 kVp images versus pixel values from Calcium(Iodine) images. Calcium(Iodine) density measurements with 4% iodine contrast are correlated with 120 kVp for calcium plaque specimens. (Based on the output equation of the model 120 kVp threshold of 130 HU as $1611\;mg/{cm}^{3}$ for Calcium(Iodine) images.)

In the first method, the 29 *ex vivo* calcium plaques shown in Fig. 1 were used to compare conventional 120 kVp HU values with DECT calcium density measurements. The linear regression analysis applied between 8924 pixel values obtained from 120 kVp images and DECT Calcium(Iodine) images in Fig. 3 gave the 120 kVp threshold of 130 HU as 1611 $mg/{cm}^{3}$ for Calcium(Iodine) images $\left(p<0.0001,R_{sq}=81.1\%\right)$.

**Figure 4:**
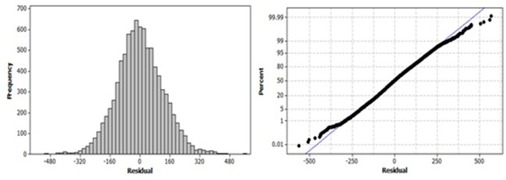
Residual plots of linear regression model. Histogram (left) of the residuals obtained by linear regression analysis performed between conventional CT HU and Calcium(Iodine) $mg/{cm}^{3}$ data. Normal probability plot (right) of the residuals obtained by linear regression analysis performed between conventional CT HU and Calcium(Iodine) $mg/{cm}^{3}$ data.

Linear regression model is based on the mean of the pixel values; however, mean of the pixel values were not the complete description of the data. Figure 3 shows that corresponding Calcium(Iodine) pixel values for 500 HU varies approximately between $1600\;mg/{cm}^{3}$ and $2200\;mg/{cm}^{3}$. For further analysis, the assumption of the residuals being normally distributed was checked by plotting the histogram and the normal probability of the residuals in Fig. 4. The assumption of residuals being normally distributed is essential for the validity of the model. The histogram showed that residuals are distributed normally. Next step to confirm the normality is to check the normal probability plot of the residuals. If the plot is an approximate straight line, then the assumption of residuals being normally distributed is valid. Normal probability of residuals was plotted versus cumulative normal percentile for a qualitative analysis to determine if the residuals were normally distributed (see Fig. 4). The normal probability plot of residuals showed a reasonably linear pattern in the center of the data; however, the tails showed departure from the fitted line. Although the histogram showed normally distributed residuals, the tails showing departure from the fitted line on normal probability plot concluded that normal distribution of the residuals was not an adequate fit for this dataset and the model was not valid. Therefore, the threshold found by linear regression model could not be used.

**Figure 5:**
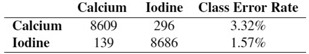
The classification table describing the agreement between actual and predicted classes of the pixels. The sum of rows is equal to the recorded calcium plaque and iodine‐enhanced blood pixels, whereas the sum of columns is equal to the predicted calcium plaque and iodine enhanced blood pixels, respectively. The third column gives the prediction error rates for calcium plaque and iodine‐enhanced blood pixels. Incorrectly classified pixels are 2.45% of the total.

The second method was based on a two‐class classification problem using pixel values recorded from Calcium(Iodine) images. A logistic regression model was trained to predict the class of pixels. Discrimination capacity of logistic regression model was measured by ten‐fold cross validation. Logistic regression model that fit the data was found to be:

(5) y=0.045x‐76.45

where *x* was the pixel value and *y* was the variable that would be used in logistic function. The output of the logistic function was the probability of pixel being calcium. Pixel value that would make the logistic regression model equal to zero gave the result of logistic function (probability of being calcium) as 0.5. The equivalent 130 HU threshold value for Calcium(Iodine) image was chosen to be the pixel value 1699 mg/cm^3^ that gave the probability as 0.5, according to the logistic regression model. Assessment of agreement between predictions and actual classes were shown in the classification table with class error rates (see Fig. 5). Total error rate was found to be 2.45%.

**Figure 6:**
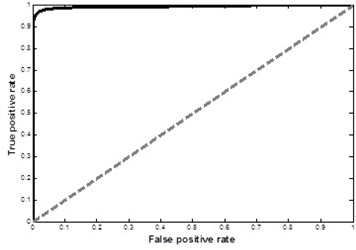
The ROC curve of the logistic regression model. The area under the ROC curve was higher than 0.9, indicating a very good discrimination which shows the sensitivity rate being high relative to the false positive rate.

Further assessment of the logistic regression model was performed by creating the relative operating characteristic curve (ROC) relating relative proportions of correctly and incorrectly classified pixels over a continuous range of probability threshold levels (see Fig. 6). Varying the probability threshold incrementally across the predicted probability range of the logistic regression model, true positive rates (sensitivity) and false positive rates were generated. Sensitivity and false positive rates were generated as in (6), (7). Positive instances are displayed as the calcium pixels on Calcium(Iodine) image.

(6) $$\text{Sensitivity}=\frac{\text{Number of positive correctly predicted}}{\text{Total number of positive instances}}$$

(7) $$\text{False positive rate}=\frac{\text{Number of positive instances incorrectly predicted}}{\text{Total number of negative instances}}$$

Discrimination capacity is described as the area under the ROC curve. A model that has no discrimination capacity will generate an ROC curve that followed the 45° line and the perfect discrimination is indicated when ROC curve follows the left hand and top axes of the unit square. The area under the ROC curve of the logistic regression model was higher than 0.9, indicating a very good discrimination which shows the sensitivity rate being high relative to the false positive rate. The area can also be interpreted as the probability that the model will correctly distinguish between calcium plaque pixels and iodine enhanced blood pixels.

### B. Preliminary patient results

**Figure 7:**
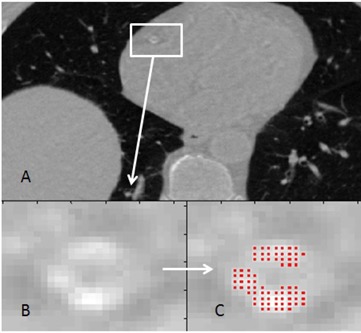
Detection of calcium plaque. Calcium(Iodine) DECT image (a) with iodine contrast administered (virtual noncontrast); RCA is located. Enlarged image of RCA (b). Pixels marked with red (c) show the detected calcium plaque in RCA.

Calcium was extensively present in the majority of these patients, including all three coronary arteries (right coronary artery (RCA), left anterior descending (LAD), and left circumflex artery (LCX)) of four cardiac patients and at least one artery in the remaining patients. Four of the patients also had calcium in their aorta and these plaques were included separately to achieve a higher overall score. For demonstration, detection of calcium plaque in RCA of one the patients using the threshold on Calcium(Iodine) image can be seen in Fig. 7. Pixels marked with red are equal or over the threshold $\left(1699\;\text{mg}/{\text{cm}}^{3}\right)$ and used in calcium scores calculations.

**Figure 8:**
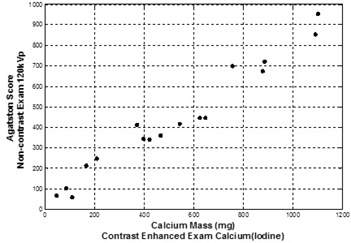
Agatston scores vs. calcium mass scores. Conventional (noncontrast) Agatston score values for patients/plaques were obtained using fast‐switched DECT with single‐energy mode. Image data for calcium mass measurements were obtained using the Calcium(Iodine) material basis pair following iodine administration obtained by fast‐switched DECT. Pearson's correlation =0.980.

**Figure 9:**
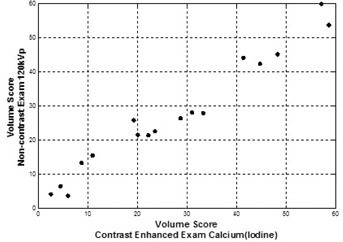
Conventional volume scores vs. DECT volume scores. Conventional (noncontrast) volume score values for same patients/plaques were obtained using fast‐switched DECT with single‐energy mode. Image data for calcium mass measurements were obtained using the Calcium(Iodine) material basis pair following iodine administration. Pearson's correlation =0.982.

Calcium mass and volume scores from contrast‐enhanced dual‐energy Calcium(Iodine) images were used to define the correlation with Agatston and volume scores obtained from noncontrast single‐energy 120 kVp images. Figure 8 compares results of measured total calcium burden obtained using DECT with administered iodine contrast to conventional noncontrast Agatston scored values on six patients on 17 vessels. Pearson's correlation coefficient between Agatston and calcium mass score was found to be 0.980. (p<0.0001) In Fig. 9, volume scores obtained from noncontrast single‐energy 120 kVp and contrast‐enhanced dual‐energy Calcium(Iodine) images are compared on the six patients on the same 17 vessels. Pearson's correlation coefficient between volume scores was found to be 0.982 (p<0.0001).

The results demonstrate that separation of calcium is possible in Calcium(Iodine) image obtained by contrast‐enhanced DECT scans. This separation allows the assessment of calcium mass in Calcium(Iodine) images as it relates to conventional Agatston and volume scores. Since calcium may not be separated from iodine contrast agent, iodine contrast‐enhanced scans showed wrongly elevated score values with conventional polychromatic images. It should be noted that exact mass density can only be understood with the application of vendor‐specific calibration factors applied to the $\text{mg}/{\text{cm}}^{3}$ density values obtained with Calcium(Iodine) images.

As presented, the preliminary patient studies demonstrated a correlation between conventional noncontrast 120 kVp Agatston scores and contrast‐enhanced Calcium(Iodine) calcium mass scores. Patient studies also indicate a correlation between noncontrast 120 kVp volume scores and contrast‐enhanced Calcium(Iodine) volume scores. Both of these correlations indicate that quantification of calcium plaque burden using a contrast enhanced DECT is possible.

Several limitations of this study are relevant.

i) Predicator variables of calcium class in logistic regression analysis were collected by recording pixel values of 29 *ex vivo* calcium plaque specimens. Using 29 *ex vivo* calcium plaque specimens might raise the question whether or not the sample size is enough to cover all possible densities of calcium found in coronary arteries. In this study, rather than finding the minimum pixel value to define calcium burden, we aimed to find the equivalent 130 HU threshold for equivalent material density units for Calcium(Iodine) image. The dataset had pixels corresponding to 130 HU and higher. The maximum pixel value that would cover the calcium plaque was void of interest for this study, since pixels having corresponding 130 HU threshold or higher values were counted to score calcium.
ii) Predicator variables of iodine‐enhanced blood class in logistic regression analysis were collected by recording pixel values of iodine enhanced blood from six patients. Small number of patients might be a concern at this point; however, the variability of actual patient data having different injection rates with two different contrast agents was part of this assessment. Also the pixels were recorded from different positions of arteries or aorta.
iii) The available number of patients for the comparison of calcium scores obtained by DECT and conventional CT limits the conclusions that calcium mass scores or volume scores obtained by DECT scans can take the place of Agatston scores or volume scores obtained by noncontrast CT scans for clinical purposes. Strong linear relation between the calcium scores obtained by contrast‐enhanced DECT and noncontrast CT is observed in 8, 9. However, this study is preliminary, and more patients who receive both conventional and noncontrast single‐energy CT scan coupled with contrast‐enhanced DECT scan need to be included to support this study.

While the proposed approach used Calcium(Iodine) as the basis pair materials, more accurate estimation of calcium burden in dual energy may be possible if the selected calcium pair was brand‐specific and not elemental iodine. Additionally, multimaterial decomposition whereby three or more materials are included in the decomposition analysis may improve the specificity for quantifying calcium. Studies have shown that full contrast material elimination can be possible when water, bone, and iodine are selected as the three basis materials when constructing the images.^(18)^ Brand‐specific material decomposition might allow better elimination of contrast material in the image and more accurate calcium scoring with contrast‐enhanced DECT.

## IV. CONCLUSIONS

This study proposed a direct measure of calcium burden by using contrast‐enhanced DECT scans to eliminate the need for an extra noncontrast X‐ray acquisition. Separation of vessels from soft tissue with noncontrast CT is not a trivial task since major tissues of the body have CT numbers ranging between −100 to 100 HU, except the bone and the lung tissue. Therefore, contrast agents such as Omnipaque, Visipaque, or Ultravist are used in routine CT imaging to enhance the contrast for better differentiability. Consequently, similar CT numbers of contrast material and the calcium plaque found in coronary arteries leads to misdiagnosis or uncertainty. Therefore, the routine tests both include contrast and noncontrast CT scans. The ambiguity of separation of calcium from contrast material on contrast‐enhanced images was solved by using virtual noncontrast images obtained by DECT. A new threshold CT number was required to detect the calcium carrying potential risk for adverse coronary events on virtual noncontrast images.

In this study, two methods were investigated to determine the 130 HU threshold for DECT scoring. An *in vitro* anthropomorphic phantom with 29 excised patient calcium plaques inserted was used for both a linear and a logistic regression analysis. An IRB approved *in vivo* prospective study of six patients was also performed to be used for logistic regression analysis. The threshold found by logistic regression model to define the calcium burden on virtual noncontrast images detects the calcium carrying potential risk for adverse coronary events correctly (2.45% error rate). DECT calcium mass and volume scores obtained by using the threshold correlates with both conventional Agatston and volume scores (r=0.98,p<0.001). Agatston scores from noncontrast conventional and contrast‐enhanced conventional scans did not demonstrate sufficient correlation for calcium quantification on CT coronary angiograms, likely due to the poor separation of calcium and iodine. The significance of this study is that a radiation dose‐neutral single DECT exam may provide both the Calcium scoring and the arterial imaging for routine cardiac assessment.
