# Erratum: Coronary calcium quantification using contrast‐enhanced dual‐energy computed tomography scans

**DOI:** 10.1120/jacmp.v16i2.5150

**Published:** 2015-01-08

## ORIGINAL CITATION

Yamak D, Pavlicek B, Boltz T, Panse P, Akay M. Coronary calcium quantification using contrast‐enhanced dual‐energy computed tomography scans. J Appl Clin Med Phys. 2013;14(3):4014.

One author of the paper, David Frakes, was mistakenly left off the author list. He should be added in the fifth spot of the author list: Yamak D, Pavlicek B, Boltz T, Panse P, Frakes D, Akay M. His affiliation is:

*School of Biological and Health Systems Engineering, Arizona State University, Tempe, AZ, USA*

